# Perceval or Trifecta to Prevent Patient–Prosthesis Mismatch

**DOI:** 10.3390/jcm9092964

**Published:** 2020-09-14

**Authors:** Daniel Hernandez-Vaquero, Carlota Vigil-Escalera, Yvan Persia, Carlos Morales, Isaac Pascual, Alberto Domínguez-Rodríguez, Emiliano Rodríguez-Caulo, Manuel Carnero, Rocío Díaz, Pablo Avanzas, Cesar Moris, Jacobo Silva

**Affiliations:** 1 Cardiac Surgery Department, Heart Area, Hospital Universitario Central de Asturias, 33011 Oviedo, Spain; carlotavel@gmail.com (C.V.-E.); cmorales@telecable.es (C.M.); diazmendezro@gmail.com (R.D.); jsilva8252@yahoo.es (J.S.); 2 Department of Cardiology, Heart Area, Hospital Universitario Central de Asturias, 33011 Oviedo, Spain; yvanpersiap@gmail.com (Y.P.); ipascua@live.com (I.P.); avanzas@gmail.com (P.A.); cesarmoris@gmail.com (C.M.); 3 Department of Cardiology, Hospital Universitario de Canarias, 38320 Tenerife, Spain; adrvdg@hotmail.com; 4 Cardiac Surgery Department, Hospital Universitario Virgen de la Macarena, 41009 Sevilla, Spain; erodriguezcaulo@hotmail.com; 5 Cardiac Surgery Department, Hospital Universitario Clinico San Carlos, 28040 Madrid, Spain; mcarnero@me.com

**Keywords:** aortic valve replacement, bioprosthesis, patient–prosthesis mismatch

## Abstract

The Trifecta aortic valve has excellent hemodynamics characteristics. Moreover, the Perceval prosthesis may achieve better hemodynamics than the conventional valves; therefore, it has been proposed to reduce the incidence of patient–prosthesis mismatch. Our aim was to compare the prevalence of this complication between both prostheses. All patients who underwent valve replacement with a Perceval or a Trifecta from 2016 to 2020 at our institution were included. We calculated the prevalence of patient–prosthesis mismatch for each prosthesis and size and performed a multinomial logistic regression model to investigate the impact of choosing one prosthesis over the other. A total of 516 patients were analyzed. Moderate mismatch was present in 33 (8.6%) in the Trifecta group and 28 (21.4%) in the Perceval group, *p* < 0.001. Severe mismatch was present in 8 (2.1%) patients with Trifecta and 5 (3.8%) patients with Perceval, *p* = 0.33. Compared with the Perceval, the Trifecta prosthesis was shown to reduce moderate patient–prosthesis mismatch: OR = 0.5 (95% CI 0.3–0.9, *p* = 0.02). Both prostheses led to a similar risk of severe patient–prosthesis mismatch: OR = 0.9 (95% CI 0.3–2.8, *p* = 0.79). Both prostheses provide a very low risk of severe patient–prosthesis mismatch. Compared with the Perceval prothesis, the Trifecta prosthesis is able to reduce by 50% the risk of moderate mismatch.

## 1. Introduction

Patient–prosthesis mismatch (PPM) is a potentially avoidable complication after aortic valve replacement (AVR). With prevalence between 2–20%, severe PPM is one of the most frequent complications and has been shown to have a detrimental impact on late survival. Some studies have shown that moderate PPM is even more frequent, with prevalence between 20% and 70% [1,2,3].

Our group and others [4,5] have recently shown that the Trifecta aortic valve (St Jude Medical Inc, St Paul, MN, USA) is likely the stented bioprosthesis with lower rates of PPM. The design of this prosthesis, different from most stented prostheses, has been suggested as responsible for the lower rate of PPM. Its small suture ring, the leaflets extended a few millimeters beyond the stent, the absence of a stitch at the top of one of its commissures, and mainly, the single sheet of pericardial tissue externally mounted on its light stent are specific characteristics of this prosthesis, which was especially designed to reduce PPM [4].

The Perceval prosthesis (LivaNova, Biomedica Cardio Srl, Saluggia, Italy) is a sutureless bovine pericardium valve mounted in a self-expandable Nilitol stent [6]. It has no sewing ring and thus, the framework of the prosthesis may be smaller than that of the conventional stented prostheses. Due to this feature, the Perceval prosthesis may achieve better hemodynamics than the conventional valves, and therefore, it has been proposed to reduce the incidence of PPM [7].

Few studies have evaluated the hemodynamic performance of the Perceval [8,9,10,11]; even fewer have evaluated the prevalence of PPM using this prosthesis [8], which is a really important measure for outcomes, and no study has compared the prevalence of this complication with the Trifecta aortic valve.

Our aim was to know (1) the prevalence of PPM for different sizes in both prostheses and (2) which of these two prostheses achieves lower rate of PPM.

## 2. Experimental Section

All patients who underwent AVR between June 2016 and June 2020 at the Hospital Universitario Central de Asturias, tertiary hospital in Spain, using the Trifecta or the Perceval prosthesis were included.

All data were prospectively collected using an electronic database. All patients underwent a transthoracic echocardiography the day before hospital discharge. These images were used for PPM evaluation. Moderate PPM was defined as the indexed effective orifice area (IEOA) ≤ 0.85 cm^2^/m^2^ and >0.65 cm^2^/m^2^. Severe PPM was defined as the IEOA ≤ 0.65 cm^2^/m^2^ [12]. Body surface area (BSA) was calculated with the DuBois formula.

### 2.1. Echocardiography

Echocardiography evaluation was performed at rest the day before hospital discharge. The effective orifice area (EOA) and other echocardiographic measures were estimated according to the American Society of Echocardiography criteria [13]. Briefly, the continuity equation was used to calculate the EOA. EOA = stroke volume/velocity–time integral thorough the prosthesis, where stroke volume is usually derived as cross-sectional area just proximal to the prosthesis multiplied by the velocity–time integral of flow at that site.

### 2.2. Surgical Procedure

Conventional full median sternotomy or mini-sternotomy was freely chosen by the main surgeon. After central cannulation, cardiopulmonary bypass, and aortic cross-clamping, the heart was protected infusing intermittent antegrade and retrograde crystalloid cold cardioplegia. The explantation of the native aortic valve was performed, and the specific measuring devices were used to select the size of the prosthesis. The Trifecta was implanted with horizontal mattress sutures with pledgets from ventricular size of the annulus to aortic surface. All Trifecta prostheses were implanted in supra-annular position. For the Perceval prostheses, three 3–0 polypropylene guiding sutures were passed at the nadir of each sinus to implant the prosthesis in the right place. After the implantation, the position of the valve was assessed and was ballooned at 4 atmospheres for 30 s. The type of prosthesis was chosen by the main surgeon.

### 2.3. Statistical Analysis

Continuous and categorical variables were described as mean ± SD and *n* (%), respectively. Continuous variables were compared using the Student’s *t* test. Previously, the robust Levene´s test was performed. Ordered categories were compared using the Mantel–Haenszel test for linear trend, and unordered categories were compared using the Fisher exact test.

Due to the observational nature of the study, and because both groups are not easily comparable, we needed to control for confounding and selection biases. To do that, we created a multinomial logistic regression analysis. PPM was defined according to IEOA. So, to control for cofounding factors, we took into account the formula of the IEOA = EOA / body surface area (BSA). The EOA depends on the following: (1) the type of prosthesis; (2) the size of the implanted prosthesis, which depends on (a) the native aortic annulus, or as an estimation, the left ventricular outflow tract diameter (LVOTD), as previously reported [4,14]; and (b) the stiffness or calcification of the native aortic annulus. This is difficult to control, but older age and diabetes have been identified as risk factors for a stiffer annulus [3,12]. Finally, the BSA is the other variable of the formula. So, the maximum model was formed by type of prosthesis, LVOTD, age, diabetes, and BSA as independent variables, and no/moderate/severe PPM was the dependent variable in a multinomial logistic regression [4].

To achieve a more parsimonious and accurate model, once this maximum model was calculated, we considered removing cofounding factors if two conditions were met: (1) the removal of that factor did not cause an important modification over the association type of prosthesis (Trifecta/Perceval) and the risk of PPM. An important modification was defined as changes in the OR > 10%. This is: (|(OR–Oradjusted)/ORadjusted| > 0.10). (2) A ≤ standard error was achieved for that OR [15]. To select the reduced model, we used the user-written command “confound” of STATA [16].

We also explored the impact of the prosthesis to prevent any degree of PPM using the usual logistic regression model and following all previously commented steps.

Linearity between covariates and logits, collinearity, and overdispersion were checked. If the assumption of linearity was not fulfilled, we made the model more flexible by categorizing into quintiles. All tests were two-sided. The group with the Perceval aortic valve was used as control or reference.

Statistical analysis was performed by using Stata software version 16 (StataCorp, College Station, TX, USA).

Our research was carried out according to The Code of Ethics of the World Medical Association (Declaration of Helsinki), and the corresponding institutional review board approved the study (ref. 68/18).

## 3. Results

### 3.1. Preoperative, Intraoperative, and Postoperative Data

A total of 549 patients underwent AVR with a Trifecta or a Perceval prosthesis during the study period at our institution. Of the prostheses that were implanted, 409 were Trifecta and 140 were Perceval. Of these 549 patients, 419 (76%) had pure aortic stenosis. The mean age was 77.1 ± 6 years and 275 (50.2%) were women. Baseline characteristics for each group are shown in Table 1.

The Trifecta group was younger (76.7 ± 5.8 vs. 78.2 ± 6.4, 0.008) and had less women (190 (46.6%) vs. 85 (60.7%)). Diabetes, body surface area, and body mass index were similar between both groups, and the Perceval group had smaller LVOTD.

Cardiopulmonary bypass and cross-clamping times were lower with the use of Perceval: 95.7 ± 37.9 vs. 81.3 ± 34.9 (*p* < 0.001) and 77.2 ± 30.3 vs. 65.3 ± 29.1 (*p* < 0.001). The in-hospital mortality, EuroSCORE II, and logistic EuroSCORE were similar. Permanent pacemaker was higher in the Perceval group: 8 (2%) vs. 15 (10.7%), *p* = 0.002. Perivalvular regurgitation ≥ II/IV was 3 (0.7%) in the Trifecta group and 8 (5.7%) in the Perceval group, *p* = 0.001. Characteristics of the operation and postoperative complications are shown in Table 2.

### 3.2. Trifecta vs. Perceval to Prevent Patient–Prosthesis Mismatch

**Figure 1:**
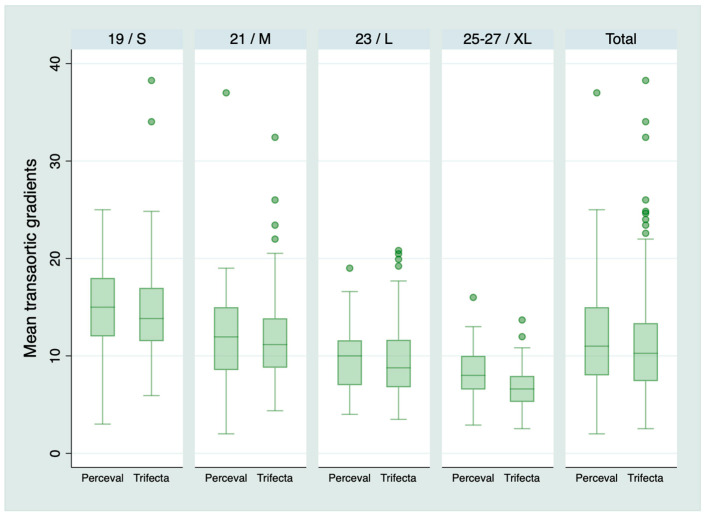
Mean transaortic gradients according to the type of prosthesis and size. Both types of prosthesis are presented together for visual inspection. However, size S of the Perceval is for an aortic annulus of 19–21 mm. Size M: 21–23 mm. Size L: 23–25 mm. Size XL: 25–27 mm [6]. Therefore, the size of a Perceval does not match the size of any Trifecta.

**Figure 2:**
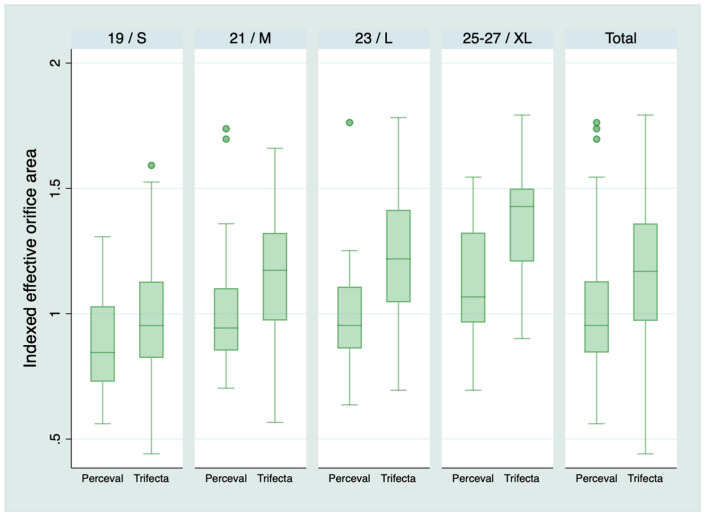
Indexed effective orifice area according to the type of prosthesis and size. Both types of prosthesis are presented together for visual inspection. However, size S of the Perceval is for an aortic annulus of 19–21 mm. Size M: 21–23 mm. Size L: 23–25 mm. Size XL: 25–27 mm [6]. Therefore, the size of a Perceval does not match the size of any Trifecta.

**Figure 3:**
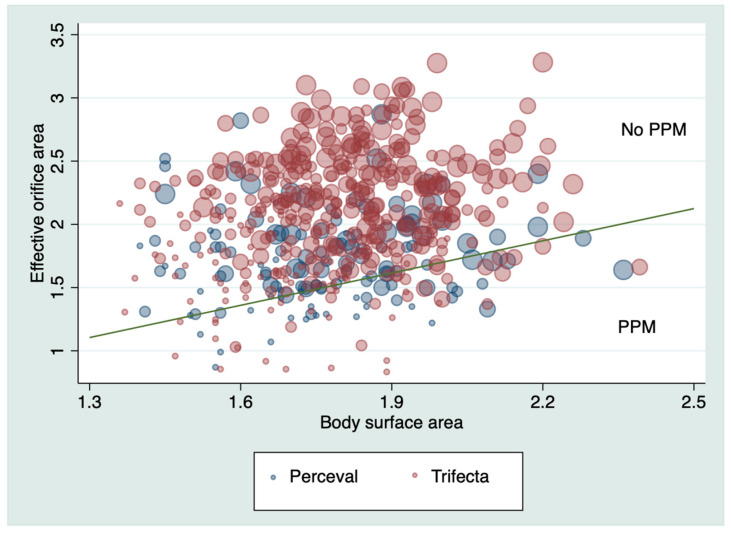
Relationship between effective orifice area and body surface area. Green line separates patients with and without PPM. The sizes of circles vary according to size of the prostheses. (19 Trifecta similar to S Perceval, 21–M, 23–L, and 25/27–XL). PPM: patient–prosthesis mismatch.

Five hundred and sixteen patients survived the operation and were discharged from the hospital: 385 patients in the Trifecta group and 131 in the Perceval group. Any degree of PPM was present in 41 (10.6%) in the Trifecta group and 33 (25.2%) in the Perceval group, *p* < 0.001. Moderate PPM was present in 33 (8.6%) in the Trifecta group and 28 (21.4%) in the Perceval group, *p* < 0.001. Severe PPM was present in 8 (2.1%) patients with Trifecta and 5 (3.8%) patients with Perceval, *p* = 0.33. Table 3 shows the prevalence of PPM for each type of prosthesis and size. Figure 1 and Figure 2 shows the mean transaortic gradient and IEOA for each prosthesis and size. Figure 3 shows the relationship between the EOA and the BSA and shows patients with and without PPM.

For the multinomial logistic regression, we explored the maximum model and selected the final model, which was formed by type of prosthesis (Trifecta vs. Perceval), LVOTD, and BSA. Compared with the Perceval, the Trifecta prosthesis was shown to reduce moderate PPM: OR = 0.5 (95% CI 0.3–0.9, *p* = 0.02), but both prostheses led to a similar risk of severe PPM: OR = 0.9 (95% CI 0.3–2.8, *p* = 0.79).

**Figure 4:**
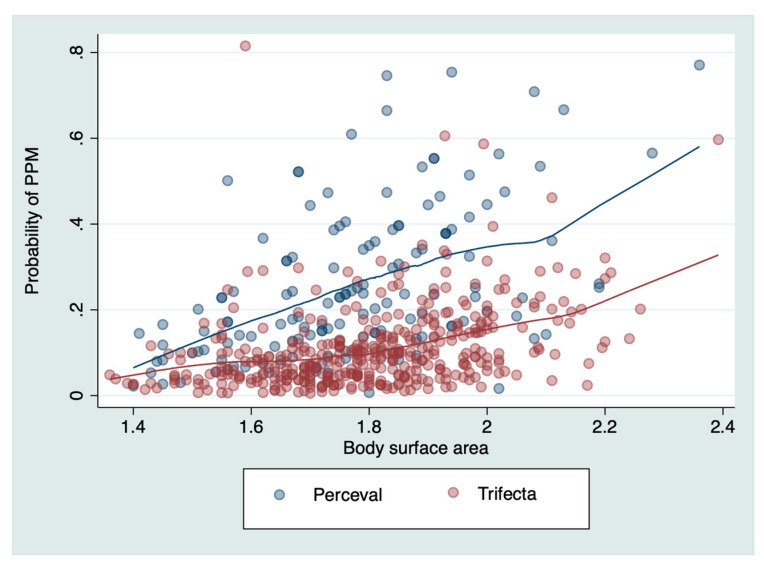
Predicted probability of PPM according to different body surface area in our sample. Lines are formed by a lowess function. PPM: patient–prosthesis mismatch.

Compared with the Perceval prosthesis, the Trifecta prosthesis was shown to reduce PPM (all degrees together): OR = 0.5 (95% CI 0.3–0.9, *p* = 0.04). Figure 4 shows the predicted probability of any degree of PPM based on the type of prosthesis and according to the different BSA for our sample.

## 4. Discussion

The main findings of our work are as follows: (1) both the Trifecta and the Perceval prostheses provide very low rates of severe PPM, and (2) compared with the Perceval, the Trifecta prosthesis is able to reduce moderate PPM.

### 4.1. Influence of Moderate PPM on Clinical Outcomes

Moderate PPM has been associated with less left ventricular mass regression [3]. This reduced regression has shown to be a predictor of worse functional class [7] and long-term survival [17]. However, unlike severe PPM, which has been shown to influence short and long-term survival [1,2,3,7], there is controversy about whether moderate PPM has an impact on clinical events. A meta-analysis formed by 58 works and more than 40,000 patients has shown that moderate PPM is a predictor of perioperative mortality [3]. This meta-analysis and another one [2] showed that moderate PPM had no impact on long-term survival. Conversely, another meta-analysis found that moderate PPM reduced long-term survival by 20% [1]. The influence of PPM seems to be more important in younger patients [2,18], women [2], patients with a low body mass index [3], and patients with left ventricular dysfunction [7,18]. The influence of moderate PPM in patients with these characteristics needs to be further investigated.

### 4.2. PPM Using the Perceval and the Trifecta

We found that for sizes ≥21 in the Trifecta or ≥M in the Perceval, severe PPM almost disappears using both prostheses. Conversely, moderate PPM is present in sizes 19–23 mm of the Trifecta and in all sizes of the Perceval. Interestingly, in this work, we show that the Trifecta prosthesis reduces moderate PPM by 50% compared with the Perceval.

Very few studies have investigated the prevalence of PPM using the sutureless valves, specifically the Perceval aortic prosthesis. In a recent multicenter prospective study, Suri et al. reported very high rates of severe PPM using the Perceval [8]. So, for the size S, the rate of severe PPM was 40%; for M and L, the rate was 30%, and for XL, it was 25%. These percentages are much higher than those previously published for conventional prostheses [1,2,3], and we do not have a sure reason for such high rates. Conversely, our work has shown that the prevalence of PPM with the Perceval is low but within the range of conventional prostheses. Moderate PPM was 21%, whereas studies have reported a range between 20% and 70% for conventional prostheses and severe PPM was 4%, whereas works have reported a prevalence between 2% and 20% [1,2,3]. Although it did not study the prevalence of PPM, a recent meta-analysis by Meco M et al. has shown lower gradients and better outcomes with the Perceval compared to usual surgical prostheses [9].

The rates of PPM using the Trifecta prosthesis are lower than those reported for any other prosthesis. Severe PPM was 2% and moderate PPM was 9%. According to the results of our work, the Trifecta prosthesis should be used when a PPM is expected or preventing any degree of PPM is a priority.

### 4.3. PPM Is Lower Using the Trifecta: Possible Reasons

There are several reasons that could explain the lower rate of PPM using the stented prosthesis Trifecta. First, the Perceval is a sutureless prosthesis and thus, it has a learning curve. We have included the first patients with Perceval at our institution. So, some degree of undersizing or oversizing could be present in the first cases. Oversizing of the Perceval has shown to increase gradients and reduce the EOA [19]. Second, despite not having a sewing ring, the Perceval has a supra-annular and intra-annular sealing collar, which occupy a space [20]. In addition, it has a stent with internally mounted leaflets. Conversely, the Trifecta has a small sewing ring, but the leaflets are externally mounted around the stent, which could increase the EOA. Third, the Perceval tissue component is based on a double sheet, whereas the Trifecta has a single sheet of bovine pericardium with no stich at the top of one leaflet commissure to facilitate the opening of leaflets. This double sheet of the Perceval could lead to less leaflet mobility, especially at rest, when the stroke volume is not very high. This prosthesis has been shown to dramatically increase its opening during the exercise [11]. Thus, our findings could change in the presence of high demand for cardiac output. 

### 4.4. Limitations

There was 3% of aortic root enlargement in the Trifecta group. Thus, these patients could have had PPM without the enlargement. In highly calcified and small aortas, the use of the Perceval prosthesis is a very good choice and may be a life-saving procedure. This article does not try to refute that. The findings of our work can only be extrapolated when both prostheses can be implanted with the same security and ease. Finally, the observational nature of the study makes confusion and selection biases possible. Only randomized controlled trials can control for unmeasured or unobserved confounding factors. We have not studied the impact of PPM during the follow-up. Based on previous meta-analyses, the lower rate of moderate PPM using the Trifecta could have a benefit in some specific groups such as young patients or those with left ventricular dysfunction. The Perceval aortic prosthesis has shown to dramatically increase its opening during the exercise [11]. Thus, our findings could change in the presence of high demand for cardiac output. Stress echocardiography could provide information on this issue, but it is beyond the scope of our work.

There is not a perfect way to detect PPM. If we take the EOA from published in vivo reference values, we are not taking into account possible variations between individuals. Geometric orifice area or in vitro area given by manufactures are used methods but are wrong measures [12,21,22]. Some experts think that the best moment for PPM evaluation is at the first visit after hospital discharge, while others think that it is at 6 months after surgery [21]. However, patients who die in this interval due to PPM will not be taken into account, and the prevalence of PPM may be undervalued [12]. Other experts proposed early during the first week after surgery or at hospital discharge, which was our method [22].

Sizes of Perceval do not match with sizes of Trifecta. For example, size S of Perceval cannot be directly compared with a 19 mm. Trifecta. Thus, we did not make any inference from a direct comparison between sizes of prostheses, and the conclusions of this paper were only drawn from the multivariate regression.

## 5. Conclusions

Both prostheses provide low rates of severe PPM. Compared with the sutureless Perceval prosthesis, the Trifecta aortic valve is able to reduce moderate PPM in regular conditions.

## Figures and Tables

**Table 1:** Baseline characteristics.

Clinical Data	Trifecta	Perceval	*p* Value
Women	190 (46.6%)	85 (60.7%)	0.004
Age, years	76.7 ± 5.8	78.2 ± 6.4	0.008
Weight, kg	75.1 ± 12.5	73.4 ± 13.7	0.17
Height, cms	161.2 ± 8.9	159.2 ± 9.4	0.03
Systemic hypertension	326 (79.7%)	107 (76.4%)	0.40
Diabetes			
Non-insulin-dependent	80 (19.6%)	32 (22.9%)	
Insulin-dependent	31 (7.6%)	14 (10%)	0.38
Dyslipidemia	220 (53.8%)	93 (63.4%)	0.01
Body surface area (m^2^)	1.8 ± 0.2	1.8 ± 0.2	0.22
Body mass index (kg/m^2^)	28.9 ± 4.5	29.1 ± 4.9	0.83
Chronic obstructive pulmonary disease	62 (15.2%)	28 (20%)	0.19
Creatinine clearance (mL/min)	63.3 ± 25.4	59.5 ± 28.5	0.17
Previous stroke	17 (4.2%)	14 (10%)	0.018
Poor mobility	8 (1.9%)	3 (2.1%)	1
Extracardiac arteriopathy	43 (10.5%)	18 (12.9%)	0.44
Previous cardiac surgery	18 (4.4%)	10 (7.1%)	0.26
Critical preoperative state	3 (0.7%)	1 (0.7%)	1
**Cardiac data**			
Active endocarditis	21 (5.13%)	6 (4.29%)	0.82
History of supraventricular arrhythmia	83 (20.3%)	26 (18.6%)	0.71
Concomitant coronary disease	140 (34.2%)	55 (39.3%)	0.48
Previous acute myocardial infarction < 3 months	10 (2.4%)	3 (2.1%)	1
Functional class NYHA			
NYHA I	20 (4.9%)	1 (0.7%)	
NYHA II	160 (39.1%)	81 (57.9%)	
NYHA III	206 (50.4%)	53 (37.9%)	
NYHA IV	23 (5.6%)	5 (0.04%)	0.005
**Echocardiographic parameters**			
Left ventricular ejection fraction			
>50%	345 (84.4%)	116 (82.9%)	
>30% y ≤50%	48 (11.7%)	16 (11.4%)	
≤30% y >20%	16 (3.9%)	8 (5.71%)	
≤20%	0 (0%)	0 (0%)	0.63
Interventricular septum > 17mm	73 (17.8%)	44 (31.4%)	0.001
Left ventricular outflow tract diameter, mm	22 ± 2.3	20.5 ± 2.4	<0.001
Systolic pulmonary pressure > 55 mmHg	48 (11.9%)	9 (6.43%)	0.004
Aortic pathology			
Pure stenosis	312 (76.5%)	107 (76.9%)	
Pure insufficiency	48 (11.8%)	8 (5.76%)	
Double lesion	48 (11.8%)	24 (17.3%)	0.046
Mean gradient, mmHg	47.3 ± 14.8	44.7 ± 14.5	0.11
Peak gradient, mmHg	79.5 ± 21.4	75.9 ± 22.9	0.16

**Table 2:** Operation characteristics and postoperative complications

Operation Characteristics	Trifecta	Perceval	*p* Value
Non-elective surgery	90 (22.3%)	33 (23.6%)	0.76
Prosthetic size			*
19 mm/S	76 (18.6%)	33 (23.6%)	
21 mm/M	151 (36.9%)	53 (37.9%)	
23 mm/L	132 (32.3%)	29 (20.7%)	
25/XL	38 (9.3%)	25 (17.9%)	
27	12 (2.9%)		
Mitral surgery	63 (15.4%)	13 (9.3%)	0.09
Tricuspid surgery	9 (2.2%)	3 (2.1%)	0.63
Proximal aortic surgery	18 (4.4%)	1 (0.7%)	0.06
Number of aortocoronary grafts			0.38
None	90 (70%)	278 (67.9%)	
1	27 (19.3%)	72 (17.6%)	
2	9 (6.4%)	38 (9.3%)	
3	6 (4.3%)	18 (4.4%)	
4 or more	0 (0%)	3 (0.7%)	
Cardiopulmonary bypass time	95.7 ± 37.9	81.3 ± 34.9	<0.001
Cross-clamping time	77.2 ± 30.3	65.3 ± 29.1	<0.001
Aortic root enlargement	12 (2.9%)	0 (0%)	0.04
EuroSCORE II	5.4 ± 5.8	4.5 ± 4.6	0.17
Logistic EuroSCORE	12.9 ± 11.3	11.3 ± 9.3	0.21
**Mortality and postoperative complications**			
Pre-discharge mortality or <30 days	24 (5.9%)	9 (6.4%)	0.47
Oro-tracheal intubation > 24 h	57 (13.9%)	23 (16.4%)	0.49
Stroke	11 (2.7%)	4 (2.9%)	1
Acute myocardial infarction	6 (4.3%)	32 (7.8%)	0.18
New need of permanent pacemaker	8 (2%)	15 (10.7%)	0.002
Supraventricular arrhythmia without effective cardioversion	18 (12.9%)	66 (16.1%)	0.42
Peri-valvular regurgitation			<0.001
I	8 (1.9%)	6 (4.3%)	
II	2 (0.5%)	5 (3.6%)	
III	1 (0.2%)	3 (2.1%)	
IV	0 (0%)	0 (0%)	
Intra-valvular regurgitation			<0.001
I	11 (2.7%)	14 (10%)	
II	1 (0.2%)	3 (2.1%)	
III	0 (0%)	0 (0%)	
IV	0 (0%)	0 (0%)	

* Size S of Perceval is for aortic annulus of 19–21 mm. Size M for 21–23 mm. Size L for 23–25 mm and size XL for 25–27 mm [6]. Therefore, the size of a Perceval does not match the size of any Trifecta. Accordingly, *p*-value was not calculated, since a direct comparison may be misleading.

**Table 3:** Prevalence of PPM and other parameters for patients at discharge.

Variables	Trifecta (*n* = 385)	Perceval (*n* = 131)
**Number 19/S**	*n* = 73	*n* = 32
Prevalence of PPM		
No PPM	54 (73.2%)	16 (50%)
Moderate PPM	13 (17.8%)	12 (37.5%)
Severe PPM	6 (8.2%)	4 (12.5%)
Mean gradient, mmHg	14.4 ± 5.1	14.6 ± 4.6
EOA, cm^2^	1.6 ± 0.4	1.5 ± 0.3
IEOA, cm^2^/m^2^	0.9 ± 0.2	0.9 ± 0.2
**Number 21/M**	*n* = 140	*n* = 52
Prevalence of PPM		
No PPM	128 (91.4%)	41 (78.9%)
Moderate PPM	10 (7.1%)	11 (21.2%)
Severe PPM	2 (1.4%)	0 (0%)
Mean gradient, mmHg	11.9 ± 4.8	12.4 ± 5.5
EOA, cm^2^	2 ± 0.3	1.7 ± 0.3
IEOA, cm^2^/m^2^	1.2 ± 0.2	1 ± 0.2
**Number 23/L**	*n* = 125	*n* = 28
Prevalence of PPM		
No PPM	115 (92%)	24 (85.7%)
Moderate PPM	10 (8%)	3 (10.7%)
Severe PPM	0 (0%)	1 (3.6%)
Mean gradient, mmHg	9.4 ± 3.9	9.9 ± 3.8
EOA, cm^2^	2.3 ± 0.4	1.8 ± 0.3
IEOA, cm^2^/m^2^	1.2 ± 0.2	1 ± 0.2
**Number 25–27/XL**	*n* = 47	*n* = 19
Prevalence of PPM		
No PPM	47 (100%)	17 (79.5%)
Moderate PPM	0 (0%)	2 (10.5%)
Severe PPM	0 (0%)	0 (0%)
Mean gradient, mmHg	6.7 ± 2.5	8.4 ± 2.9
EOA, cm^2^	2.5 ± 0.4	2.1 ± 0.2
IEOA, cm^2^/m^2^	1.4 ± 0.2	1.1 ± 0.2

EOA: effective orifice area. IEOA: indexed effective orifice area. PPM: patient–prosthesis mismatch. Size S of Perceval is for aortic annulus of 19–21 mm. Size M for 21–23 mm. Size L for 23–25 mm and size XL for 25–27 mm [6]. Therefore, the size of a Perceval does not match the size of any Trifecta. Accordingly, p-value was not calculated since a direct comparison can be misleading.

## References

[1] Head S.J., Mokhles M.M., Osnabrugge R.L., Pibarot P., Böhm M., Takkenberg J.J.M., Bogers A.J.J., Kappetein A.P. (2012). The impact of prosthesis–patient mismatch on long-term survival after aortic valve replacement: A systematic review and meta-analysis of 34 observational studies comprising 27,186 patients with 133,141 patient-years. Eur. Heart J..

[2] Chen J., Lin Y., Kang B., Wang Z. (2013). Indexed effective orifice area is a significant predictor of higher mid- and long-term mortality rates following aortic valve replacement in patients with prosthesis-patient mismatch. Eur. J. Cardio Thorac. Surg..

[3] Dayan V., Vignolo G., Soca G., Paganini J.J., Brusich D., Pibarot P. (2016). Predictors and outcomes of prosthesis-patient mismatch after aortic valve replacement. JACC Cardiovasc. Imaging.

[4] Hernández-Vaquero D., Díaz R., Pascual I., Rozado J., De La Hera J.M., Leon V., Avanzas P., Martín M., Iglesias D.G., Calvo D. (2018). The prevalence of patient-prosthesis mismatch can be reduced using the trifecta aortic prosthesis. Ann. Thorac. Surg..

[5] Tadokoro N., Fukushima S., Shimahara Y., Matsumoto Y., Yamashita K., Kawamoto N., Minami K., Kobayashi J., Fujita T. (2018). Trifecta vs. Magna for Aortic Valve Replacement―Differences in Clinical Outcome and Valve Hemodynamics. Circ. J..

[6] Sorin Group (2015). Perceval Sutureless Aortic Heart Valve. Instructions for Use.

[7] Bilkhu R., Jahangiri M., Otto C.M. (2019). Patient-prosthesis mismatch following aortic valve replacement. Heart.

[8] Suri R.M., Javadikasgari H., Heimansohn D.A., Weissman N.J., Ailawadi G., Ad N., Aldea G.S., Thourani V.H., Szeto W.Y., Michler R.E. (2019). Prospective US investigational device exemption trial of a sutureless aortic bioprosthesis: One-year outcomes. J. Thorac. Cardiovasc. Surg..

[9] Meco M., Montisci A., Miceli A., Panisi P., Donatelli F., Cirri S., Ferrarini M., Lio A., Glauber M. (2018). Sutureless Perceval aortic valve versus conventional stented bioprostheses: Meta-analysis of postoperative and midterm results in isolated aortic valve replacement. J. Am. Heart Assoc..

[10] Shrestha M., Fischlein T., Meuris B., Flameng W., Carrel T., Madonna F., Misfeld M., Folliguet T., Haverich A., Laborde F. (2015). European multicentre experience with the sutureless Perceval valve: Clinical and haemodynamic outcomes up to 5 years in over 700 patients. Eur. J. Cardio Thorac. Surg..

[11] Rubino A.S., Biancari F., Caruso V., Lavanco V., Privitera F., Rinaldi I., Sanfilippo M., Millan G., D’Urso L.V., Castorina S. (2017). Hemodynamic assessment of Perceval sutureless bioprosthesis by dobutamine stress echocardiography. Echocardiography.

[12] Hernández-Vaquero D. (2015). Patient prosthesis mismatch in adult congenital heart disease. Heart.

[13] Zoghbi W.A., Chambers J.B., Dumesnil J.G., Foster E., Gottdiener J.S., Grayburn P.A., Khandheria B.K., Levine R.A., Marx G.R., Miller F.A. (2009). Recommendations for evaluation of prosthetic valves with echocardiography and doppler ultrasound. J. Am. Soc. Echocardiogr..

[14] Ugur M., Suri R.M., Daly R.C., Dearani J.A., Park S.J., Joyce L.D., Burkhart H.M., Greason K.L., Schaff H.V. (2014). Comparison of early hemodynamic performance of 3 aortic valve bioprostheses. J. Thorac. Cardiovasc. Surg..

[15] Maldonado G., Greenland S. (1993). Simulation Study of Confounder-Selection Strategies. Am. J. Epidemiol..

[16] Doménech J.M., Navarro J.B. (2020). Find the Best Subset for Linear, Logistic and Cox Regression: User-Written Command Confound for Stata.

[17] Ali A.A., Patel A., Ali Z., Abu-Omar Y., Saeed A., Athanasiou T., Pepper J. (2011). Enhanced left ventricular mass regression after aortic valve replacement in patients with aortic stenosis is associated with improved long-term survival. J. Thorac. Cardiovasc. Surg..

[18] Price J., Toeg H., Lam B.-K., Lapierre H., Mesana T.G., Ruel M. (2014). The impact of prosthesis–patient mismatch after aortic valve replacement varies according to age at operation. Heart.

[19] Cerillo A.G., Amoretti F., Mariani M., Cigala E., Murzi M., Gasbarri T., Solinas M., Chiappino D. (2018). Increased Gradients after Aortic Valve Replacement with the Perceval Valve: The Role of Oversizing. Ann. Thorac. Surg..

[20] Folliguet T.A., Laborde F., Zannis K., Ghorayeb G., Haverich A., Shrestha M. (2012). Sutureless Perceval Aortic Valve Replacement: Results of Two European Centers. Ann. Thorac. Surg..

[21] Pibarot P., Dumesnil J.G. (2012). Valve Prosthesis–Patient Mismatch, 1978 to 2011. J. Am. Coll. Cardiol..

[22] Daneshvar S.A., Rahimtoola S.H. (2012). Valve prosthesis-patient mismatch (VP-PM): A long-term perspective. J. Am. Coll. Cardiol..

